# Measurement of the quadriceps (Q) angle with respect to various body parameters in young Arab population

**DOI:** 10.1371/journal.pone.0218387

**Published:** 2019-06-13

**Authors:** Ramada R. Khasawneh, Mohammed Z. Allouh, Ejlal Abu-El-Rub

**Affiliations:** 1 Department of Basic Medical Sciences, Faculty of Medicine, Yarmouk university, Irbid, Jordan; 2 Department of Anatomy, Faculty of Medicine, Jordan University of Science and Technology, Irbid, Jordan; 3 Department of Physiology and Pathophysiology, University of Manitoba, Winnipeg, Canada; Cleveland Clinic, UNITED STATES

## Abstract

The quadriceps angle (Q angle), formed between the quadriceps muscles and the patella tendon, is considered clinically as a very important parameter which displays the biomechanical effect of the quadriceps muscle on the knee, and it is also regarded a crucial factor for the proper posture and movement of the knee patella. The Q angle is routinely and regularly used as an assessment parameter during the diagnosis of many knee-related problems, including the anterior knee pain, osteoarthritis, and degenerative knee disorders. This study had been conducted so as to measure the normal Q angle values range in the Arab nationalities and determine the correlation between Q angle values and several body parameters, including gender, height, weight, dominant side, and the condylar distance of the femur. The study includes 500 healthy young Arab students from the Yarmouk University and Jordan University of Science and Technology. The Q angle of those volunteers was measured using a universal manual Goniometer with the subjects in the upright weight-bearing position. It was found that Q angle was greater in young women than young men. Also, the analysis of the data revealed an insignificant increase in the dominant side of the Q angle. In addition, the Q angle was significantly higher in the taller people of both sexes. However, the Q angle did not present any considerable correlation with weight in the study population; conversely, it was clearly observed that there was a link with the condylar distance of the femur in both sexes. It was also noticed that the Q angle increased remarkably when there was an increase in the condylar distance. Consequently, it turned out that the gender, height, and the condylar distance were momentous factors that had impact on the Q angle in our study samples. However, weight and dominancy factors did not show to have any influence on the values in our study.

## Introduction

The Q angle, which is also known as quadriceps angle, is defined as the angle formed between the quadriceps muscles and the patella tendon. It was described for the first time by Brattstrom in 1964 [1]. It is an evident medical fact that the measurement of the Q angle is a very decisive indicator of the biomechanical function in the lower extremity since this measurement reflects the effect of the quadriceps mechanism on the knee, it also gives an idea how the thigh muscles function to make the knee moves, as well as how the knee patella tracks in the groove of the knee joint [2,3]. Moreover, Q angle has become accepted as an important factor in assessing knee joint function and determining knee health in individuals suffering from an anterior knee pain [2–4]. When it is assessed correctly, it will supply very useful information concerning the alignment of the pelvis, leg, and foot [5–7]. It is beyond doubt that misalignment will cause problems to the knee function. Therefore, the determination of the Q angle is particularly momentous for patients who are athletically and physically active [8]. Furthermore, it is essential to measure the angle of female patients who walk for health purposes, climb stairs frequently, or participate in a regular form of sports [5,9].

The literature of the documented values of Q angle by various researchers vary. It is well-known that the normal Q angle should fall between 12 and 20 degrees; the males are usually at the low end of this range; while females tend to have higher measurements [6,10–13]. Other researchers’ suggestions that the values should be as low as 10 degrees reflect problems. Recently, some studies have illustrated that values between 8° and 10° for men and up to 15° for women are deemed normal, but values which are higher than those can indicate an abnormality. Davies and Larson have not stated a range for normal values, but they regarded Q angles >20° as excessive [14]. The measurement of Q angle is usually deemed excessive when it increases the lateral pull of the quadriceps femoris muscle on the patella and potentiates patellofemora disorders [2,15].

An excessive Q angle indicates a tendency for added biomechanical stress during repetitive activities using the knee [2] because it interferes with the smooth movement of the patella in the femoral groove [2,3]. Over the passage of time, especially with sports activities, it will cause muscle imbalance [16] and eventually wearing away of the cartilage on the underside of the patella which can be translated into the loosing of the articular surface of the knee [17]. Therefore, the resultant damage is permanent which makes the complete recovery after treatment impossible.

Moreover, excessive Q angle leads to excessive pronation of the foot, and the increase of the pronation time will cause excessive internal rotation of the tibia which will change the quadriceps mechanism and lateral tracking of the patella [18]. Eventually, the more rapid progression from knee dysfunction to patellofemoral arthralgia can be developed into degenerative joint disease. Controlling the foot pronation can often reduce the detrimental effects of an abnormal Q angle [19].

In a nutshell, this study was undertaken to investigate the influence of gender, weight and height and leg dominance on Q-angle utilizing a goniometer with the subject standing on a weight bearing position. In addition to identifying any interrelation between the Q angle and the femur condylar, the study is designed to further investigate the mean Q angle in the Arab countries including, some Gulf Countries population with the goal of making the data be used and compared to the values of other parts of the world as well as to help improving the clinical diagnosis and assessment of the misalignments of the knee joint.

## Materials and methods

### Study sample

The subjects for the study were normal healthy adult students from Yarmouk University and the Jordan University of Science and Technology. The students with a history of trauma, fractures, or dislocation in the lower limbs were excluded from the study. Also, participants with musculoskeletal pathology, that could influence the Q-angle were excluded from the study. The Q angle measurements had been performed bilaterally for each volunteer. The total study sample consisted of 500 volunteers (100 Jordanians, 100 Palestinians, 100 Syrians, 100 Saudis, 50 Kuwaitis and 50 Omanis (with ages ranging from 19 to 25 years. Among the study subjects, 267 were females and 233 were males.

### Measurement procedure

**Fig 1:**
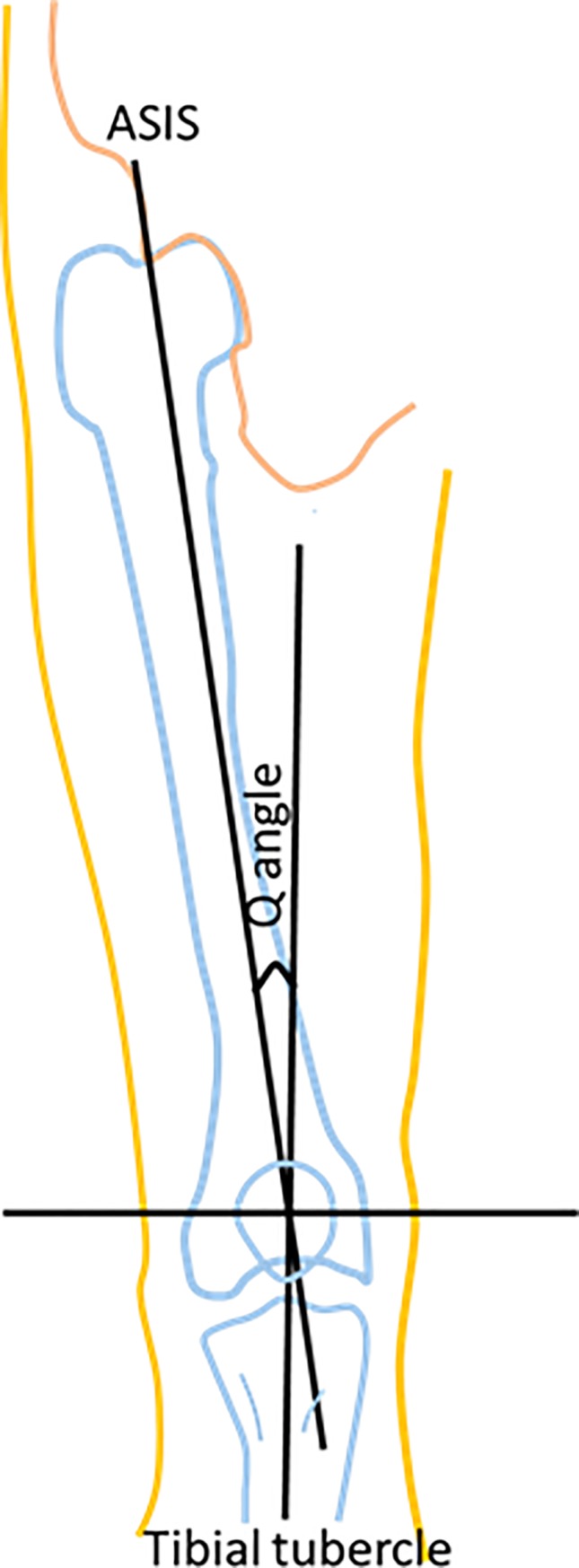
Q angle and marker locations: Anterior Superior Iliac Spine (ASIS) and tibial tuberosity.

Measurement procedures were performed after securing the approval of the Institutional Research Board at JUST (IRB-# 34-120-2019) (S1 Fig). An appropriate written consent report was distributed before embarking on the measurements (S2 Fig). In addition, a brief description of the procedure was demonstrated to make it familiar to the subjects after recording their nationalities, age, gender, weight, height, and dominant side on a specific investigation paper sheet. Also, the determination of the leg dominance was based on their individual preference when being asked to kick a ball. The Q angle was measured with a full circle universal manual goniometer which is made of clear plastic with the subject standing in the erect weight-bearing position. The anterior superior iliac spine (ASIS), the midpoint of the patella, and the tibial tuberosity were replaced and determined. The hinge of the goniometer was located at the midpoint of the patella, the goniometer arms were adjusted to become positioned to the line joining the ASIS and the line joining the tibia tuberosity, then the small angle on the goniometer was read as the Q angle (Fig 1). Both sides were measured for each individual. Each side was measured 3 times, and the mean value of the angle was calculated.

A manual caliper, scaled from 0 cm to 20 cm and with a marginal error of ± 1 mm, was used to measure the condylar distance of the femur for both sides of each volunteer. The subject first stood in the anatomical position with the feet facing forward, and the leg was flexed to 90° with the result that the femoral condyles became prominent and easily palpable at that position. After the fixed arm of the caliper was placed on the lateral condyle, and the movable arm was then adjusted to the medial condyle; the condylar distance measurement for each side had been determined and recorded on the participant’s investigation sheet.

After collecting the requested information and measurements, the data were transferred into a computer to perform the required statistical analysis.

### Statistical analysis

After applying the Levene test to determine the homogeneity of variance, the data were evaluated by one-way analysis of variance (ANOVA) or independent samples *t*-test at 0.05 and 0.01 levels of significance. The Scheffe *post hoc* analysis test was performed when it was needed to examine statistical differences between the groups when necessary. The data were presented as mean ± standard error of the mean (SEM).

## Results

### Variation in Q angle with sex

**Fig 2:**
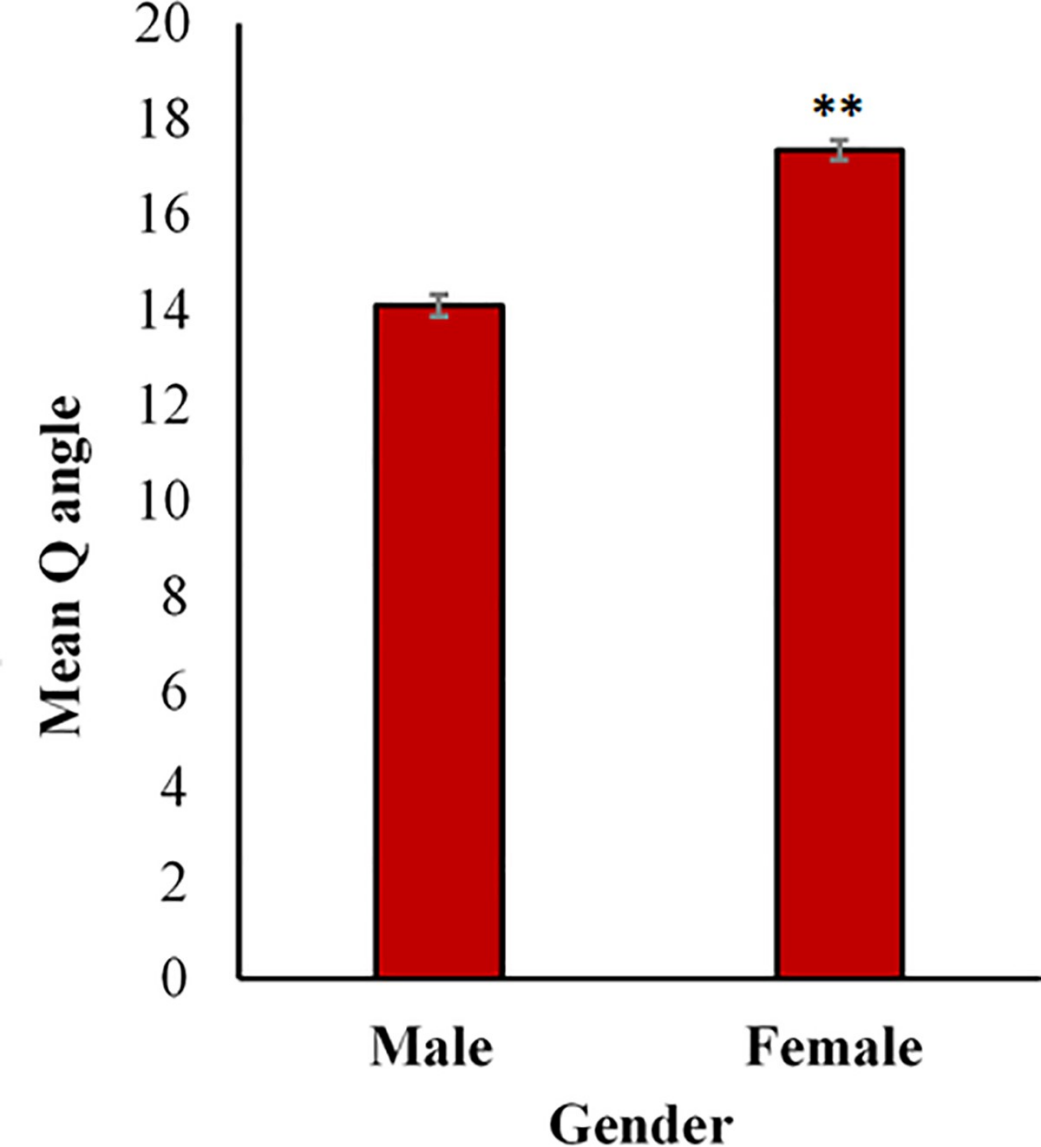
Variation of the Q angle with sex in adult population. The data revealed a remarkable difference in the Q angle between males and females with higher values in females. Each column represents the mean Q angle ± standard error of the mean (SE). **P<0.01 (t-test).

The volunteers were divided according to sex as follows: male (n = 233) and female groups (n = 267). The Q angle in both sides was significantly (P<0.01) greater in the female subjects than in the male subjects, such a finding indicated that the Q angle was more prominent in the female subjects than their counterparts in the male subjects. The mean Q angle ± SEM in the female subjects was 17.35 ± 0.225 ^o^, whereas that in the male subjects was 14.1 ± 0.21^o^ (Fig 2).

### Variation in Q angle with height and weight

**Fig 3:**
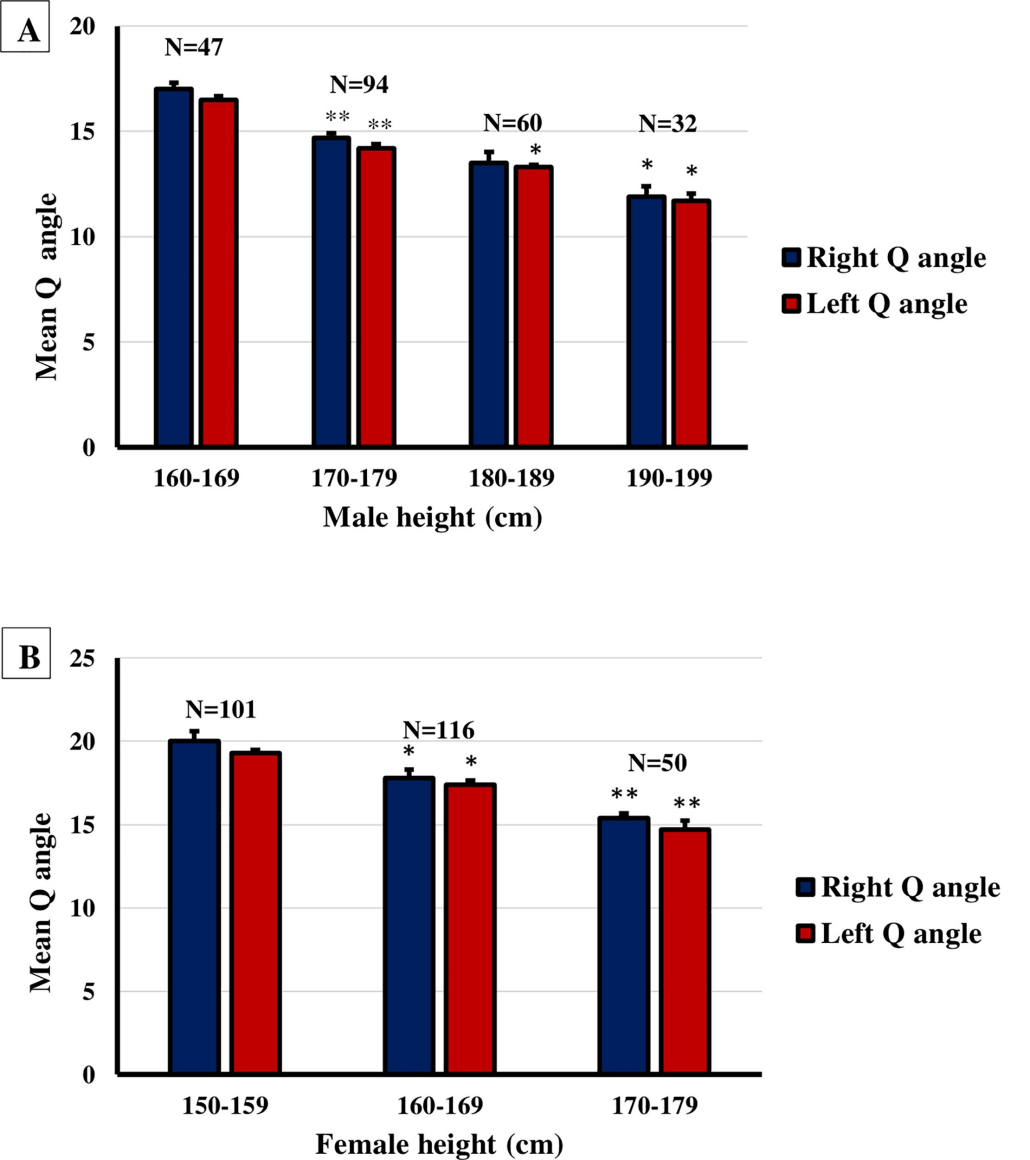
**Variation of Q angle with respect to height in males (A) and females (B).** A columnar representation for the relationship between the mean Q and the height of in males (A) and in females (B). Values are mean Q angle ± standard error (SE). There is a significant increase in Q angle as the condylar distance increases in both sides. *P<0.05, **P<0.01.

A sample of 500 adults aged between 18 and 25 years was divided in line with sex as follows: males 233 and females 267. Each category was studied separately and independently so as to determine the variation in Q angle with respect to height and weight. The male subjects were divided into 4 groups according to their heights with each the height interval of each group was 10cm. It turned out that a significant (*P*<0.05) variation in Q angle with height was observed in both sides of the male subjects (Fig 3A). The female subjects were divided into 3 groups in accordance with their height, with each group consisted of a 10-cm interval. It was also found that a considerable (*P*<0.05) variation in Q angle with height was observed in both sides of the female subjects (Fig 3B).

The male subjects were divided into 5 groups on the basis of their weights. Each group included a weight interval of 10 kg. It was interesting to find that no significant (*P*>0.05) variation in Q angle with weight was observed in both sides of the male subjects (Table 1).

**Table 1:** Measurements of the Q angle with respect to weight in the adult men.

Weight (kg)	Right Q angle	Left Q angle	P value (χ^2^)
60–69 N = 32	13.6 ± 0.23°	13 ± 0.51°	0.62
70–79 N = 57	14.2 ± 0.42°	13.9 ± 0.33°	
80–89 N = 83	14.2 ± 0.16°	14 ± 0.29°	
90–99 N = 32	14.5± 0.71°	14.2 ± 0.11°	
100–109 N = 29	15 ± 0.11°	14.6 ± 0.20°	

The female subjects were also divided into 5 groups in agreement with their weights with each group had a weight interval of 10 kg. It was astonishing to find that there was also no important (*P*>0.05) variation in Q angle with weight was observed in both sides of the female subjects (Table 2).

**Table 2:** Measurements of the Q angle with respect to weight in the adult women.

Weight (kg)	Right Q angle	Left Q angle	P value (χ^2^)
40–49 N = 36	16.9 ± 0.66°	16.6 ± 0.02°	0.58
50–59 N = 86	17.4 ± 0.12°	16.8 ± 0.56°	
60–69 N = 90	17.5 ± 0.25°	17 ± 0.38°	
70–79 N = 40	17.7± 0.45°	17.2 ± 1.1°	
80–89 N = 15	18.5 ± 0.80°	18 ± 0.19°	

### Variation in Q angle with the dominant side

**Fig 4:**
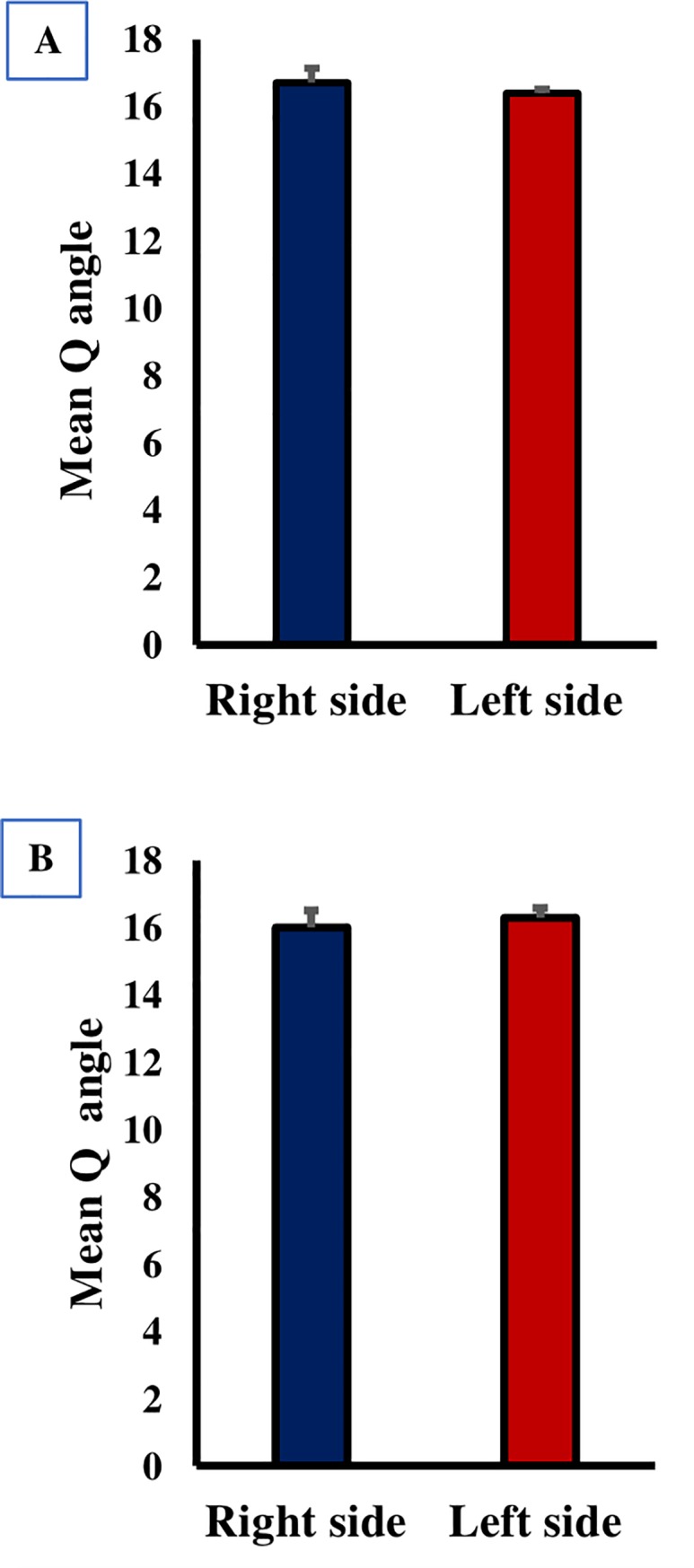
Variation of the Q angle with dominant side in adult population. (A) right side dominant volunteers showed no significant differences. (B) left side dominant volunteer with the results displayed no significant difference. Each column represents the mean Q angle ± standard error of the mean (SE).

The Q angle was measured in a sample of 437 right-side dominant volunteers with the remarkable result that was no significant (P>0.05) between the Q angle measurement of the right and left side in both sexes in the right side-dominant volunteers. Normally speaking, the Q angle value on the right side is more often greater than the left. The mean Q angle ± SE was 16.7 ± 0.43° in the right side and 16.4 ± 0.12° in the left side (Fig 4A).

The Q angle was measured in a sample of 63 left-side dominant volunteers. There was also, no significant (P>0.05) between the Q angle measurement of the right and left side in both sexes in the left side dominant volunteers. The Q angle value on the left side is more often greater than the left. The mean Q angle ± SE was 16.0 ± 0.51° in the right side and 16.3 ± 0.28° in the left side (Fig 4B).

### Variation in Q angle with the condylar distance

**Fig 5:**
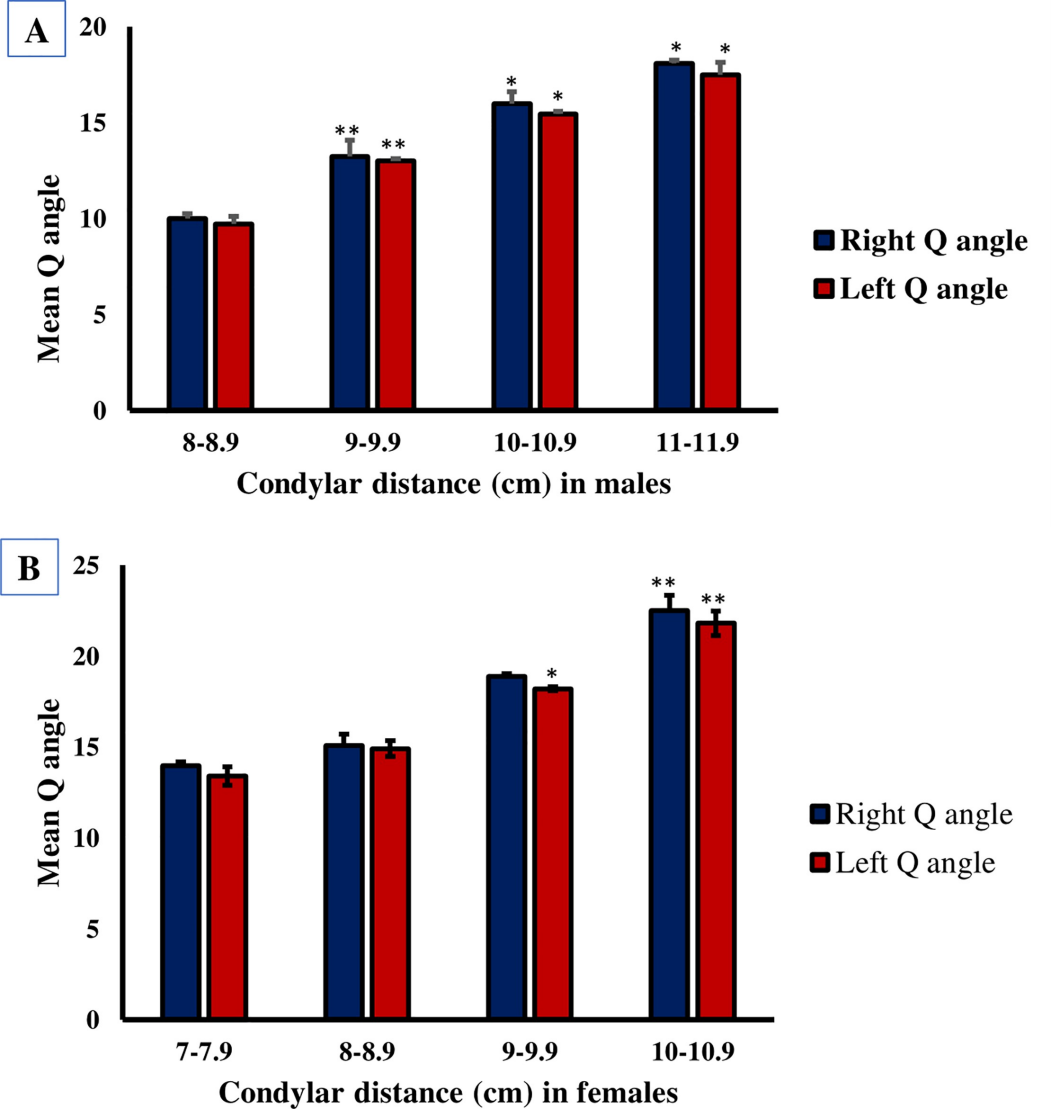
**Variation of Q angle with condylar distance in males (A) and females (B).** A columnar representation for the relationship between the mean Q and the condylar distance of the femur in males (A) and in females (B). Values are mean Q angle ± standard error (SE). There is a significant increase in Q angle as the condylar distance increases in both sides. *P<0.05, **P<0.01.

A sample of 489 adult volunteers, whose age range was between 18-25 years, were divided into four groups which was based on the length of their right condylar distance. The right Q angle was measured and compared among those four groups. We found that the Q angle and the condylar distance was directly proportional, the Q angle (*P*<0.05) increased significantly as the condylar distance increased (Fig 5A).

Additionally, the left Q angle was measured and compared among the same four groups. We, also came to the same conclusion, we found that the Q angle (*P*<0.05) insignificantly increased as the condylar distance increased (Fig 5B). Our results indicated that the Q angle is directly correlated with the condylar distance of the femur in the study group regardless of which side is measured.

## Discussion

The Q angle (The quadriceps femoris angle) is one of the most clinically used parameter in evaluating the quadriceps forces and factors acting on the patellofemoral joint which is considered to be as an indicator for sports performance as well as in the diagnosis of several patellofemoral painful disorders and diseases. Knee alignment indicators such as Q angle are highly correlated with the quadriceps femoris muscularity. Any alteration in alignment that increases the Q angle is thought to increase the lateral force on the patella. This can be harmful because the increase in this lateral force may lead to increase the compression of the lateral patella on the lateral lip of the femoral sulcus. In the presence of a great enough lateral force, the patella may actually sublux or dislocate over the femoral sulcus when the quadriceps muscle is activated on an extended knee. It has also been found that an abnormal Q angle may also influence neuromuscular response and quadriceps reflex response time [20], consequently, it may be a risk factor for anterior cruciate ligament injury [21].

The aim of this study was to pinpoint the relationship between the Q angle and various body parameters. Numerous studies on Q angle have been conducted worldwide aimed to correlate the variations in the Q angle values to the variations in race [11,12,22]. The present study provides new findings about the Q angle and its relation to several body parameters in Arab countries population.

The outcomes of this study, which revealed that Q angle was greater in women compared to men, were similar to earlier reported results regarding the variations in Q angle with gender that was higher in females as well [13,22]. In our study, we made use of the goniometer to assess the absolute difference in Q angle between young men and young women which turned out to be 3.25° higher in females than males. Interestingly, the values of the Q angle in both sexes in Arab population were relatively higher than what had been reported in other countries and ethnicities [12]. On the other hand, the mean value in this study appears to be close to the values reported by Clifford [23]. The possible explanation of females having high Q angle values can be attributed to the fact that their pelvis anatomy is wider than males’ pelvis which is extrapolated by having a long distance between the pelvis and the patella in comparison to the distance from the patella to the tibial tuberosity, thereby inducing an alternation in the position of the anterior superioriliac spine that has a huge impact on the Q angle values [24]. These explanations are contrary to what was previously reported by Jaiyesimi, A.O. and Jegede, O.O’s studies (2009) which suggested that the difference in the Q angle between the males and females maybe ascribed to the fact that men tend to be taller than women, and that the Q angle is usually slightly smaller in the taller persons [10]. The higher Q angle values in females increase the articulating surfaces compression which is clinically important in elucidating the fact regarding why females are at higher risk of patellofemoral pain. Recent studies have found that high Q-angle values in females are also linked to the increase in cartilage thickness measurements of the medial femoral condyle and cartilage grading in female patients of osteoarthritis. The Q angle values in Arab females, measured in the current study, are greater than the normal values range reported in other countries and ethnicities, therefore, the Arab females tend to be at greater risk of developing knee abnormalities. The outcomes of our study further confirmed what was previously discovered regarding the fact that Q angle is significantly smaller in taller person on both sexes. Moreover, previous studies had shown that the quadriceps contraction had a considerable corollary on the Q angle values by affecting the patella position [25,26]. Generally speaking, the fact that males are more physically active than females lead to lower Q angle values as a result of their stronger quadriceps muscle.

Based on the findings of the present study, the Q angle values do not vary significantly with the weight of the study population. Sra A. *et al* (2008) also reported no noticeable variation in the Q angle with weight [27].

Relatively speaking, few worldwide studies have focused on Q angle bilateral variability. In the present study, the Q angle was greater on the dominant side compared with the non-dominant side, but this difference was not statistically significant. Hahn and Foldspang were among the first researchers to make a detailed study of the bilateral variability in the Q angle [8]. Following this study, other studies have documented similar bilateral variations [12,13,27,28] with only two studies found that this bilateral differences significantly affected the Q angle [27,28].

To further investigate the Q angle, the condylar distance was measured in both legs using a digital caliper. This has been the first study that investigates the relationship between the Q angle and the condylar distance of the femur. The results show a significant increase in the Q angle as the condylar distance increases in both sexes. The correlation between Q angle and condylar distance is clinically important in the diagnosis of degenerative arthritis and other knee degenerative abnormalities.

## Supporting information

S1 Fig A student's approved form for conducting the Q angle research. (PDF)

S2 Fig The ethical approval of the Institutional Research Board (IRB) at Jordan University of Science and Technology to conduct the Q angle research. (DOCX)
