# Supplementary material for: Measurement of the quadriceps (Q) angle with respect to various body parameters in young Arab population
Source: PLoS One. 2019 Jun 13;14(6):e0218387. doi: 10.1371/journal.pone.0218387 (PMC6564690; doi:10.1371/journal.pone.0218387)
Supplement: S1 Fig — (PDF) [file pone.0218387.s001.pdf]

نموذج موافقه الطالب على اجراء بحث علمي

اسم الطالب:

الجنسيه:

الجامعة:

انا الموقع ادناه اوافق على المشاركه في البحث الموسوم ب " دراسة زاوية العضله رباعية الرؤوس المتشكلة عند مفصل الركبة البشري" و المشرفه عليه الدكتوره رمادا خصاونه من قسم التشريح, كليه الطب, جامعه اليرموك. و الهدف منه هو معرفه مقدار التغير في زاويه العضله رباعية الرؤوس بالنسبه الى عوامل الجنس, الطول, الوزن, و العرق.

قبل موافقتي على اعلامي من قبل الباحث بمايلي:

1. موافقه الجهات المعنيه على اجراء البحث.
2. اهداف البحث
3. كيفيه اجراء القياسات من قبل الباحث
4. الحالات التي يمكن للباحث فيها ايقافي عن المشاركه بالبحث
5. كيفيه الحفاظ على سريه المعلومات الخاصه بالمشارك

المشاركه في هذا البحث اختياريه, فلن يعاقب الطالب في حال قرر عدم المشاركه.

في حال توقيعك على هذا المستند فأنت تقر بأنك توافق اختياريا على المشاركه في هذا البحث و ان المعلومات المذكوره اعلاه قد شرحت لك بالكامل من قبل الباحث.

التاريخ:

اسم المشارك:

توقيع المشارك:

التاريخ	اسم الشاهد الاول:	التوقيع:
التاريخ	اسم الشاهد اللثاني:	التوقيع:

المشرف على البحث: د. رمادا خصاونه

A student's approved form for conducting a scientific research

Student name:--

Nationality:---

University name:---

I, the undersigned, agree to participate in the research entitled "Measurement of the Quadriceps (Q) Angle with Respect to Various Body Parameters ", supervised by Dr. Ramada Khasawneh, Department of Anatomy, Faculty of Medicine, Yarmouk University. The goal of this study is to know the amount of change in the Q angle relative to factors of sex, height, weight, dominant side, and intercondylar distance

Before accepting I was informed by the researcher by the follows:
1. the research obtained the acceptance from the RIB
2. Research objectives
3. How to make measurements by the researcher
4. The cases in which the researcher can stop the volunteer from participating in the research
5. How to maintain the privacy of the participant's information

The participant in this research is optional, the student will not be punished if he/she decides not to participate.

If you sign this document, you agree to participate in this research and that the above information has been fully explained to you by the researcher.

Date:
Participant Name:
Signature of the Participant:

date	Name of the first witness:	Signature
date	Name of the second witness:	Signature

Research supervisor: Dr. Ramada Khasawneh
