# Supplementary material for: Measurement of the quadriceps (Q) angle with respect to various body parameters in young Arab population
Source: PLoS One. 2019 Jun 13;14(6):e0218387. doi: 10.1371/journal.pone.0218387 (PMC6564690; doi:10.1371/journal.pone.0218387)
Supplement: S2 Fig — (DOCX) [file pone.0218387.s002.docx]

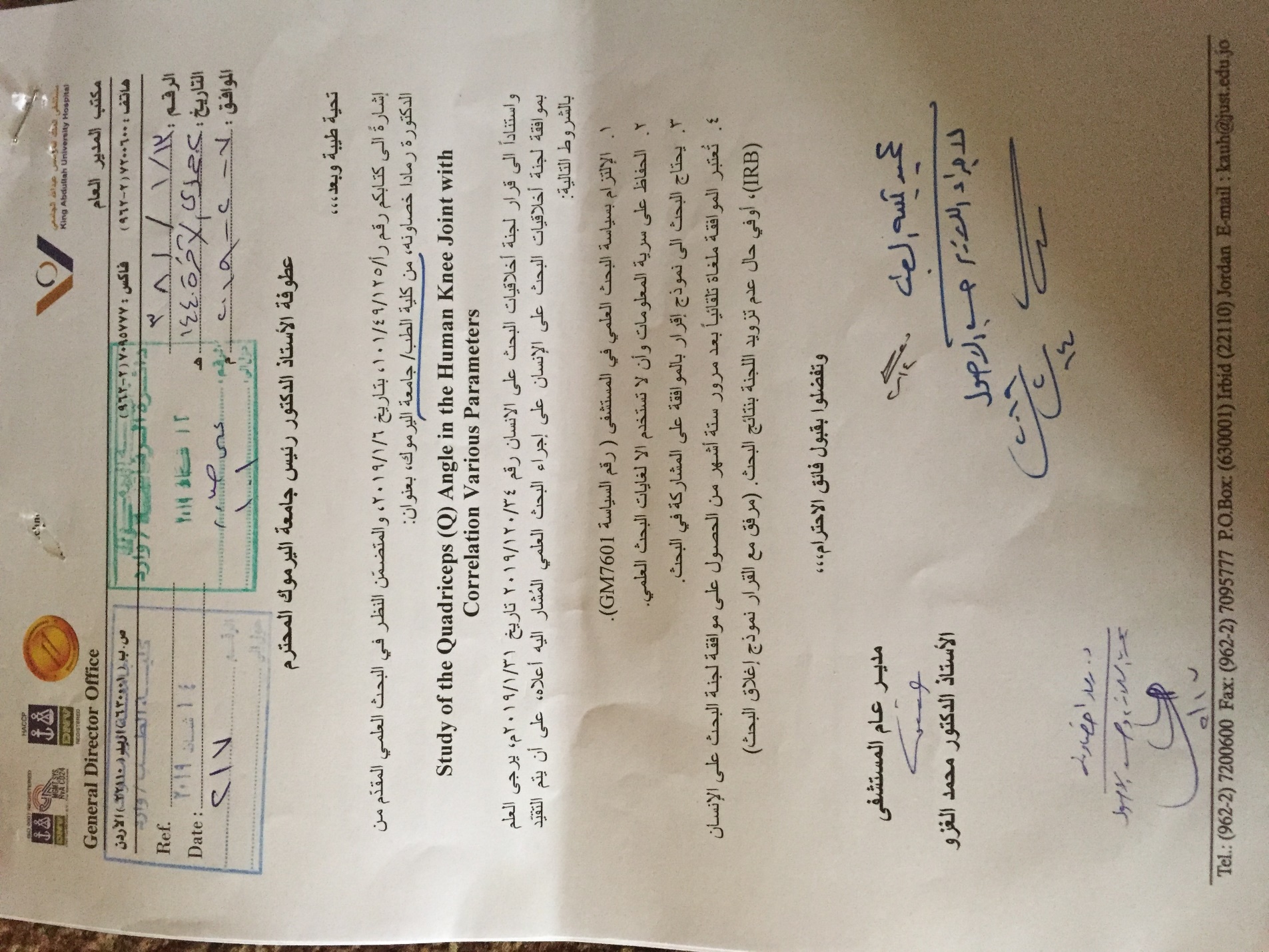


President of Yarmouk University

Good greeting:

Reference to your book number RA/125/49/101, in 6/1/2019, included consideration of research Submitted by Dr. Ramada Khasawneh from the Faculty of Medicine / Yarmouk University, entitled:

**Study the Quadriceps (Q) angle in the Human Knee Joint with Correlation to Various Parameters**

Based on the decision of the Ethics Committee for Human Research number 34/120/2019, date 31/1/2019, Please be informed that it is approved by the Ethics Committee for Human Research to carry out the research referred to above. The following conditions are met during the research:

1. Commitment to its research policy in the hospital (Policy No GM7601)
2. To preserve the confidentiality of information and to be used only for the purposes of scientific research
3. The research needs a form of approval to agree to participate in the research
4. The approval shall be canceled after six months of obtaining the approval of the Ethics Committee for Human Research (IRB)or in the case of non-approval to provide the committee with the results of the research

Please accept our serious greeting

Director General of the hospital

Dr. Mohammed Al-Gazo.
